# Immunoexpression and prognostic role of p53 in different subtypes of epithelial ovarian carcinoma

**DOI:** 10.7555/JBR.26.20110103

**Published:** 2012-04-24

**Authors:** Lihong Chen, Lianxiang Li, Feng Chen, Dalin He

**Affiliations:** a Department of Cancer Research/Key Laboratory of Environment and Gene Related to Diseases of Ministry of Education, Xi'an Jiaotong University, School of Medicine, Xi'an, Shaanxi 710061, China;; b Department of Gynecology and Obstetrics, Shaanxi Provincial People's Hospital, Xi'an, Shaanxi 710068, China;; c Department of Pharmacology, Vascular Biology Center, Medical College of Georgia, Augusta, GA 30912, USA.

**Keywords:** ovarian cancer, p53, prognosis

## Abstract

We sought to investigate the significance of p53 expression for epithelial ovarian carcinoma. In this study, we used immunohistochemical method to investigate the expression patterns of p53 in different subtypes of epithelial ovarian carcinoma. We found that the expressions of p53 protein in epithelial ovarian cancer (pituita, serosity and intima) were 88.9%, 75% and 100%, respectively, while the recurrence rates among three cancer subtypes were significantly different (33.3%, 12.5% and 0%, respectively; *P* < 0.05). Compared with patients without lymph node metastasis, the expression of p53 in patients with lymph node metastasis was significantly strong (68.75% and 100%, respectively; *P* < 0.05). However, the recurrence rate in the patients with lymph node metastasis (40%) was higher than that without lymph node metastasis (6.25%, *P* < 0.05). The expressions of p53 protein in ovarian cancer between I-II (25%) stage and II-IV stage (100%) were significantly different (*P* < 0.05), and the recurrence rates between the two groups were significantly different (0% and 31.25%, respectively, *P* < 0.05). Therefore, p53 protein has an intimate relationship with the malignant degree and the prognosis of ovarian cancer.

## INTRODUCTION

The incidence of ovarian cancer ranks third among all gynecologic cancers, behind cervical and endometrial cancer, but it ranks first in mortality[1]. In the United States, ovarian cancer is the fifth most common cancer among women, with approximately 16,090 deaths and 4,000 new cases per year. The 5-year survival rate for ovarian cancer is only 20%-30% with a long-term chemotherapy after surgery[2]. However, the molecular mechanisms that lead to the development of ovarian cancer are unknown. The activations of many oncogenes have been implicated in the onset of ovarian cancer. P53 is a tumor suppressor protein which regulates the expression of various genes involved in apoptosis, growth arrest, inhibition of cell cycle progression, cell differentiation and DNA repair or senescence in response to genotoxic or cellular stress[3]. Recently, many studies have suggested that in the progression of ovarian cancer, the alteration of genes on chromosome 17 may frequently be of significance[3]-[6]. Since *p53* is located on chromosome 17p13.1, we aimed to determine the expressions of p53 in epithelial ovarian cancer.

In the present study, we used immunohistochemical technique to investigate the expression pattern of p53 in epithelial ovarian carcinoma, and, furthermore, we explored the relationship between prognostic factor and clinicopathological features of p53 in different types of epithelial ovarian carcinoma.

## SUBJECTS AND METHODS

### Clinical samples

Between January 2005 and April 2006, 26 consecutive epithelial ovarian carcinoma patients without any previous treatments were selected in this study. Each patient underwent surgery as part of treatment for epithelial ovarian cancer at the Shaanxi Provincial People's Hospital. All of the patients received chemotherapy following primary surgical exploration. All patients were followed up. The definition of recurrent ovarian cancer is that after satisfactory cytoreductive surgery, regular and adequate chemotherapy, ovarian cancer has returned less than 6 months after stopping chemotherapy. Evidence and signs of recurrent ovarian cancer include increased levels of CA125, physical examination revealed tumor, and imaging study revealed tumor, pleural effusion and unexplained intestinal obstruction. Once two of the criteria mentioned above have been satisfied, ovarian cancer recurrence should be considered. This study was approved by related institutional review boards of the Shaanxi Provincial People's Hospital, and all of the participants signed the informed consent.

### Antibodies and reagents

Mouse anti-human p53 monoclonal antibody was purchased from B&D Biosciences Pharmingen (San Diego, CA, USA). SP kit was purchased from Zhongshan Golden Bridge Biotechnology Co., Ltd. (Beijing, China).

### Immunohistochemical analysis

Specimens were fixed in paraformaldehyde, embedded in paraffin, cut at 5 µm, and then respectively detected with hematoxylin and eosin (H&E) and immunohistochemical staining. Samples were deparaffinized in xylene at room temperature for 30 min, rehydrated in graded ethanol and washed in phosphate buffer saline (PBS). The samples were then placed in 10 mol/L citrate buffer (pH 6.0) and boiled in a microwave for epitope retrieval for 10 min. Endogenous peroxidase activity was quenched by incubating tissue sections in 3% H_2_O_2_ for 10 min. Sections were incubated with mouse anti-human p53 monoclonal antibody that was at 1:25 dilution in a humidity chamber at 37°C for 30 min. They were washed in PBS for 5 min at room temperature. Sections were subsequently stained according to the labeled streptavidin peroxidase method using a commercial SP kit and were visualized using 3,3′-diaminobenzidine (DAB). Sections were then followed by counterstaining the sections with hematoxylin. Sections from epithelial ovarian carcinoma tissue with known p53 expression were used as positive control. Sections incubated without the primary antibody were used as negative control. For the assessment of staining, examination fields were randomly selected. The cells at each intensity of staining were recorded on a scale of negative (no staining cell), positive (staining cells of <50%, or weak staining), and strong positive (staining cells of >or =50%, or strong staining).

### Statistical analysis

All the data were analyzed using the SPSS software 16.0 for Windows (SPSS Inc. Chicago, IL, USA). The χ^2^-test was used to evaluate the expression of p53 protein in ovarian cancer. *P* < 0.05 was regarded as the threshold value for statistical significance.

## RESULTS

**Fig. 1:**
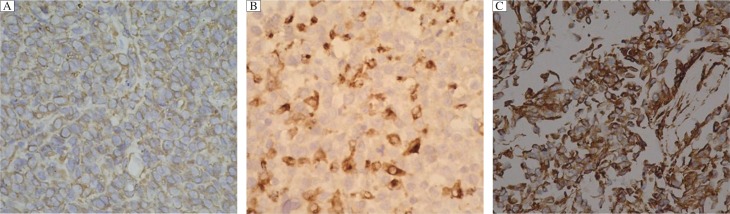
The expressions of p53 in the three subtypes of ovarian cancer. Tissue samples were stained by immunohistochemistry. A: negative control of p53. B: positive expression of p53 (+). C: strong positive expression of p53 (++).

The expressions of p53 protein in different pathological epithelial ovarian cancer subtypes (pituita, serosity and intima) were 88.9%, 75% and 100%, respectively, However, the recurrence rates among the three subtypes were obviously significant (33.3%, 12.5% and 0%, respectively; *P* < 0.05). In patients with lymph node metastases, the expression of p53 was high (100%), while it was low in patients without lymph node metastasis (68.75%). In addition, the recurrence rate of ovarian cancer patients (40%) with lymph node metastasis was significantly higher (*P* < 0.05) than patients without lymph node metastasis (6.25%) (***Table 1*** and ***Fig. 1***).

**Table 1:** The expression pattern of P53 protein in different subtypes of ovarian caner

Group	*n*	-	+	++	Positive rate		Recurrence [*n*(%)]
					(+)	(++)	
Mucinous tumor	9	0	1	8	100%	88.90%	3(33.30%)
Serous tumor	16	0	4	12	100%	75%	2(12.50%)
Endometrioid tumor	1	0	0	1	100%	100%	0(0%)
Lymph node metastases	10	0	0	10	100%	100%	4(40%)
No lymph node metastases	16	0	5	11	100%	68.75%	1(6.25%)

Positive staining of p53 was seen in 8 (53%) of 15 early stage cancers (stage I/II, and in 11 (100%) of 11 advanced stage cancers (stage III/IV). Statistically significant difference was found between the two groups (*P* < 0.01). Furthermore, the recurrence rates in epithelial ovarian cancer patients between early stages and late stages (I/II *vs* III/IV) were significantly different (0% and 31.25%, respectively; *P* < 0.01) (***Table 2***).

## DISCUSSION

Despite significant advances in chemotherapy, the prognosis of epithelial ovarian cancer remains poor. Apart from clinicopathological parameters associated with prognosis, p53 is one of the well-known molecular markers[4]–[6]. P53 has been described as “the guardian of the genome”, referring to its role in conserving stability by preventing genome mutation. P53 is a critical tumor suppressor that maintains genetic stability in mammals by playing multiple roles in cell cycle arrest, apoptosis, senescence and differentiation[10]. Therefore, p53 could prevent the passage of DNA damage to daughter cells. More and more evidence indicates that the critical tumor suppressor p53 is mutated in over half of all human cancers, including ovarian cancer. Accumulating evidence has indicated that *p53* mutation, including the hotspot mutations (R175H, R248W and R273H)[11],[12], not only lose the tumor suppression activity of widetype *p53*, but also gain novel oncogenic activities to promote tumorigenesis and drug resistance[13],[14]. The expressions of p53 mutants are more stable and correlated with poor prognosis of cancer patients[15]–[17].

Even though many investigators have demonstrated that *p53* mutations are associated with advanced disease and poor prognosis in ovarian cancer, the prognostic significance of *p53* mutation remains a controversial issue[18]. In ovarian cancer, some studies have shown the prognostic effectiveness of *p53* mutants, while other studies did not show that[19]–[23]. However, in our study we found that mutation of *p53* correlated with some clinicopathological parameters including advanced stage and lymphatic metastasis.

There are some limitations to our study. For example, we did not investigate gene mutation of *p53* and immunohistochemistry, which may have led to false-negative or false-positive findings. Some investigators have reported that null mutations or homozygous gene deletion of p53 showed negative immunostaining. In addition, it has been reported that highly positive p53 staining and totally negative p53 staining predicted poor survival for ovarian cancer. Despite these limitations, immunohistochemistry has shown some advantages of being an easy procedure, highly cost effective and a stable status of samples in a clinical setting. The present study evaluated the expression of p53 mutants in ovarian cancer patients treated with surgery and postoperative chemotherapy. In conclusion, expression of p53 mutants is closely correlated with malignancy and prognosis of epithelial ovarian carcinoma, so it could be considered as a prognostic indicator.

**Table 2:** The expression patterns of p53 protein in different stages of ovarian caner

Group	*n*	-	+	++	Positive rate		Recurrence [*n*(%)]
					(+)	(++)	
Stage I∼II	10	0	8	2	100%	25%	0(0%)
Stage II∼IV	16	0	0	16	100%	100%	5(31.25%)
